# The First Interlaced Continuum Robot, Devised to Intrinsically Follow the Leader

**DOI:** 10.1371/journal.pone.0150278

**Published:** 2016-02-25

**Authors:** Byungjeon Kang, Risto Kojcev, Edoardo Sinibaldi

**Affiliations:** 1 Center for Micro-BioRobotics, Istituto Italiano di Tecnologia, Pontedera, Italy; 2 The BioRobotics Institute, Scuola Superiore Sant’Anna, Pontedera, Italy; Politehnica University of Bucharest, ROMANIA

## Abstract

Flexible probes that are safely deployed to hard-to-reach targets while avoiding critical structures are strategic in several high-impact application fields, including the biomedical sector and the sector of inspections at large. A critical problem for these tools is the best approach for deploying an entire tool body, not only its tip, on a sought trajectory. A probe that achieves this deployment is considered to follow the leader (or to achieve follow-the-leader deployment) because its body sections follow the track traced by its tip. Follow-the-leader deployment through cavities is complicated due to a lack of external supports. Currently, no definitive implementation for a probe that is intrinsically able to follow the leader, i.e., without relying on external supports, has been achieved. In this paper, we present a completely new device, namely the first interlaced continuum robot, devised to intrinsically follow the leader. We developed the interlaced configuration by pursuing a conceptual approach irrespective of application-specific constraints and assuming two flexible tools with controllable stiffness. We questioned the possibility of solving the previously mentioned deployment problem by harnessing probe symmetry during the design process. This study examines the entire development of the novel interlaced probe: model-based conceptual design, detailed design and prototyping, and preliminary experimental assessment. Our probe can build a track with a radius of curvature that is as small as twice the probe diameter, which enables it to outperform state-of-the-art tools that are aimed at follow-the-leader deployment. Despite the limitations that are inherently associated with its original character, this study provides a prototypical approach to the design of interlaced continuum systems and demonstrates the first interlaced continuum probe, which is intrinsically able to follow the leader.

## Introduction

The development of flexible tools that can be safely deployed to hard-to-reach targets while avoiding (or minimizing contact with) critical structures is strategic in many application fields, including the biomedical sector [1–2] and the sector of inspections at large [3].

In the biomedical sector, for instance, the quest for flexible yet controllable tools that can be steered through soft tissue and cavities has intensified. The problem of guiding needle-like tools over a selected trajectory through soft tissue is commonly known as needle steering [4] because the tool tip–tissue actions are used to steer the needle while advancing. Because the needle is embedded in tissue, its proximal sections travel to locations that have already been passed by the distal sections; thus, the needle maintains its pose up to tissue deformation. Accurately following a given trajectory is challenging due to tissue deformation, and due to the variability of local tissue properties. Needle steering is an active research field, with valuable contributions that encompass advanced control and tool design [5–8]. Following a chosen trajectory through a cavity is challenging due to a lack of supports. In addition, tool flexibility must be frequently negotiated with functionality (ideally, the tool should be flexible for deployment yet stiff for operation), which requires that the tool stiffness be modulated [9]. This applies to both articulated and continuum devices; several groups are developing tools with controllable stiffness, which is primarily based on jamming [10–11] and active friction control (by tensioning cables [12], pressurization [13], or vacuum creation in the case of interlocked structural components [14]).

We address the problem of deploying the entire tool body, not only its tip, on a selected trajectory. Regardless of the specific application field, a track should be incrementally created to achieve this deployment through a cavity so that tool deployment reduces to a track-building problem. Track-building can be either virtually or physically achieved [9]. In the former case, the tool features a certain number of segments that are actively steered during insertion to ideally assume the same pose in the correspondence of the same trajectory point. This strategy is dependent on suitable sensors (e.g., for the insertion depth) and the storage of the tool tip trajectory information in a memory system. Physical track-building can be addressed by using coaxial tool guides that are inserted and rigidified, starting from the outer one, to telescopically build the track (telescoping method). Another option envisages a couple of tool guides to be alternately inserted and rigidified to support each other and build the track (alternating method). After successful deployment, the tool follows the trajectory that is incrementally traced by its tip. For an articulated tool, all segments follow the distal section and the tool is considered to achieve follow-the-leader (FTL) deployment (or to follow the leader) [15]; this terminology also applies to continuum tools. We focus on the problem of achieving FTL deployment with a tool that moves through a cavity.

Currently, a robotic colonoscope is being developed that employs a control strategy that is similar to virtual track-building: it is a modular probe that encompasses both flexible and rigid segments; each module can bend by pulling wires [16]. It is devised to advance into the colon; due to its control strategy, it manages to exert less force on the tubular cavity walls. However, it is also dependent on this contact to support track-building; thus, it cannot pursue FTL deployment irrespective of the cavity boundary and accurately follows a given trajectory. The considered probe features an outstanding design and properly serves its purpose. A tendon-driven colonoscope that was capable of propagating a distal shape toward the proximal sections while advancing was commercially available [17]; however, it is not detailed in the recent literature.

Physical track-building has been recently addressed using concentric tube robots [18]. These continuum tools do not allow for stiffness modulation, and the tubes are inherently coupled by their telescopic arrangement; thus, FTL deployment is not feasible in general. However, concentric tube robots can follow the leader in a few special cases by introducing some restrictions on the actuation mechanism [18]. In these cases (for which no material torsion occurs, which is consistent with previous heuristics [19]), the FTL capability is not dependent on external supports (i.e., it is intrinsic) because it is hard-coded by design in the geometric and material properties of the tubes. Moreover, physical track-building was addressed by the articulated probe introduced in [12], which features two coaxial mechanisms composed of rigid cylindrical links that are serially connected by ball-socket joints. The stiffness of each mechanism can be increased by pulling tendons that run through the entire probe (to increase the friction of the ball-socket joints), and track-building can be intrinsically achieved by the alternating method. However, the articulated nature of the considered tool can constrain the length of the deployment steps and limits the maximum curvature during deployment (due to limitations on the joint angle [20]). To the best of our knowledge, the FTL capabilities of the considered probe are not quantitatively assessed in the literature. However, it likely represents the best embodiment achieved so far for an FTL tool, and it has been developed to perform surgical robotics tasks [21].

Based on these findings, a definitive implementation for an FTL probe has not been achieved. However, an FTL probe can significantly impact key market sectors, such as the market for flexible endoscopes and visual inspection equipment. The former was estimated at $2.4 billion in 2010 and is forecasted to increase at a compound annual growth rate (CAGR) of 4% to attain $3.1 billion by 2017 [22–23]. The latter was estimated at approximately $297.6 million in 2011 and is expected to increase at an 8.1% CAGR to contribute 21.6% of the total nondestructive inspection equipment market by 2016 [24]. A probe that effectively achieves FTL deployment would be rewarding, and the expected impact motivates the development of novel approaches.

We addressed the FTL problem from a conceptual perspective. We questioned the possibility of developing a continuum probe that is intrinsically able to follow the leader, without relying on special operating conditions. We addressed a continuum tool based on its enhanced potential to smoothly conform to a given trajectory compared with articulated tools [25], also based on some remarkable devices under development, such as the device in [26] (whose segments are coupled by the telescopic arrangement and are not conceived for deployment). We assumed two continuum devices (also referred to as robots, which is consistent with the literature) with controllable stiffness, and we invoked symmetry to derive an embodiment for the alternating track-building method. Our approach was essentially grounded on the idea to use the design constraints introduced by symmetry to simplify the probe concept and pave the way for its effective development. We addressed a basic design problem by disregarding application-specific constraints at an early stage.

The main contribution of this study is as follows: motivated by the FTL problem and by pursuing the previously mentioned conceptual approach, we present a completely new device, namely, the first interlaced continuum robot [27]. This study encompasses model-based conceptual design, detailed design and prototyping, and the preliminary experimental assessment of the novel interlaced probe. We focused on the deployable tool and only neglected the actuation unit design (because it can be implemented through classical linear stages [12,28–30]); for this reason, we primarily employed the term probe when describing the novel device. We anticipate that our results demonstrated FTL capabilities that are beyond the state of the art and validated our concept and approach.

We first present the probe design and prototyping. In particular, we also address the conceptual design for a fundamental understanding of the proposed device. Moreover, we also recall the main results of the mathematical model that was introduced to solve the track-building problem; details are included in S1 Text. We then present the experimental assessment. Finally, after discussing the main achievements and limitations of the proposed device, we add some concluding remarks.

## Concept, Model-Based Design and Fabrication

### Concept and model-based design ice-breaking

**Fig 1:**
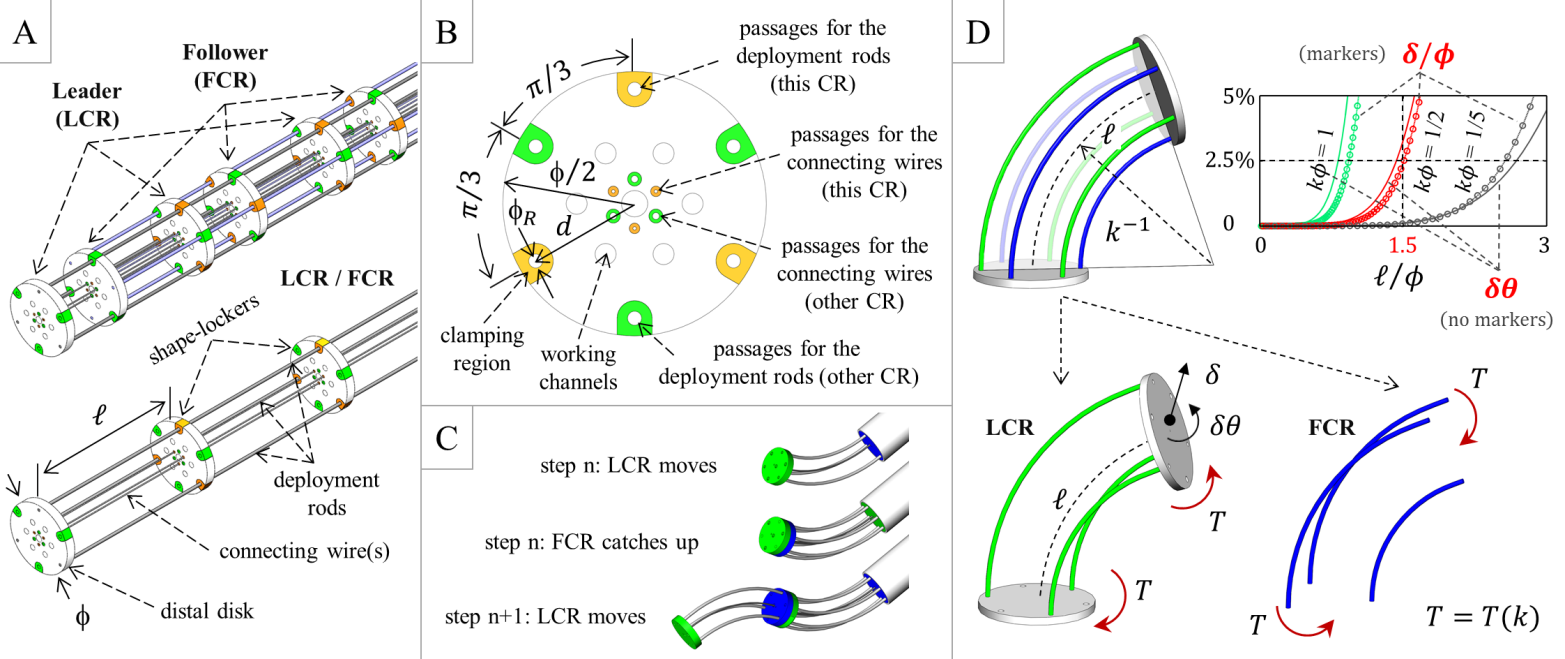
Probe concept and model-based design ice-breaking. (A) Schematic of the probe, which consists of two interlaced continuum robots (CRs); the probe diameter ϕ, the span ℓ, and the main components of each robot are indicated. (B) Shape-locker concept, which highlights the strong symmetry that underpins the interlaced configuration: each shape-locker must enable the simultaneous operation of both CRs. (C) Example steps of the FTL deployment. (D) Model-based span determination based on an idealized probe section that builds a circular track. We determined the load-free pose of the leader (locked, depicted in green in the figure) that approximates the circular shape; we subsequently applied the torque T that is associated with the bent rods of the follower (unlocked, depicted in blue in the figure) and examined the resulting deviations (linear δ and angular δθ) from the sought shape. For the selected curvature κ, we obtained the maximum ℓ that can be employed to approximate the circular shape with a 5% relative error (top-right graph): based on the targeted curvature κϕ = 1/2, we selected ℓ/ϕ = 1.5.

Fig 1A–1C shows the probe concept. The probe features two interlaced continuum robots (CRs) that are identical; they are concentrically located and angularly shifted and can slide on each other. The CRs are devised to alternately guide each other, with the exception of the distal section of the distal CR, which is free to incrementally build the deployment track. Thus, the proximal CR systematically follows the distal CR during deployment: there is a leader CR (LCR) and a follower CR (FCR); the CRs reverse their role during retraction.

Each CR features a (passive) distal disk; n shape-lockers (active disks), three deployment rods, and three connecting wires (for ease of prototyping, we subsequently utilized only one connecting wire). The distal disk and the shape-lockers are fastened to the connecting wires, and the wire span between two adjacent disks (denoted by ℓ in Fig 1A) is constant. The rods are only fastened to the distal disk: when the shape-lockers are activated (CR in the locked configuration), they are also fastened to the rods; when the shape-lockers are not activated (CR in the unlocked configuration), the rods can locally slide relative to the shape-lockers. While unlocked, each CR is deployed by pushing its rods: steering is achieved by differential pushing, and the shape-lockers are pulled by the connecting wires that systematically operate under traction. Conversely, CR retrieval is primarily performed by pulling the connecting wires (that pull the shape-lockers), and differential rod pulling also supports retrieval. After activation, all shape-lockers simultaneously clamp the deployment rods, and each CR segment (segment between two adjacent disks) can achieve a certain curvature based on the lengths of the corresponding rod segments. This action enables a three-dimensional (3D) pose for the entire CR. We assume that the shape-lockers are thin and do not excessively constrain the CR movement/pose. We also assume that the CR bending stiffness is primarily determined by the rods: the contribution of the connecting wires (thin and located near the ideal probe centerline) is negligible. We nonetheless assume that the bending stiffness of the wires is not null; thus, their deformed shape can approximate the ideal probe centerline, which is useful for probe deployment.

The shape-locker concept (Fig 1B) accounts for the simultaneous operation of both CRs; it also permits the passage of the rods/wires of “the other CR”. The interlaced configuration imposes a hexagonal symmetry (which was only slightly reduced during prototyping), which yields the possible values of ± π/3 and π rad for the angular shift between the two CRs. Additional working channels respect symmetry, and electrical wires can be hosted, e.g., in the central passage (or near the ideal centerline to minimize possible stretching), or within tubes that replace the connecting wires.

Probe deployment is obtained by repeated steps (Fig 1C). At each step, the unlocked LCR protrudes onto the locked FCR to incrementally build the pursued track. Shape-locking is subsequently switched, and the unlocked FCR catches up by protruding onto the locked LCR. The protrusion length can be modulated (the span ℓ provides an upper bound) based on the local track curvature, and the steps can be iterated based on the number n of the active segments (the CRs are initially encased, and the maximum deployment length can be affected by the rod guiding system). By symmetry, the control law for deployment of the FCR is related to the control law for deployment of the LCR (they are not identical due to the angular shift). Because the CRs are identical, track-building can be achieved if and only if the stiffness change between the unlocked configuration and the locked configuration is sufficiently large. Retraction is performed by reversing the deployment steps and consistently pulling the connecting wires. By pulling the rods with the reversed control law, possible perturbations to the probe shape can be minimized.

For the probe to achieve FTL deployment, its geometrical and material properties must be selected to effectively perform track-building. Thus, we addressed design ice-breaking by developing a mathematical model that provides the span that permits to build a track portion with the curvature κ (for fixed values of the remaining design parameters). The model is fundamental for this study; however, it is detailed in S1 Text because its formulation and solution strategy deserve a level of detail that cannot be properly framed in this paper. The modeling approach and the results are presented in the following section.

The probe design was initialized by selecting the diameter ϕ (characteristic probe size, refer to Fig 1A) and the deployment rods. We selected ϕ = 30 mm to pave the way for the detailed design of miniaturized shape-lockers without introducing technological challenges that are incommensurate with the ice-breaking stage. We selected superelastic NiTi rods (Euroflex, Pforzheim, Germany) to confer enhanced elasticity to the probe (Young modulus E = 58 GPa; Poisson’s ratio ν = 0.33 [31–33]). We adopted ϕ_R_ = 0.8 mm (rod diameter), and we selected d = 14 mm (distance from the disk center, refer to Fig 1B) to maximize the bending stiffness (through the area moment of inertia of the rods [34]). We subsequently addressed the following problem: how to build a circular track portion with the given curvature κ by deploying the idealized probe section shown in Fig 1D. We targeted the curvature κ_des_ such that κ_des_ϕ = 1/2 to outperform the state of the art. We determined the static pose of the section using a two-step simplified approach: (i) first, we deployed (and locked) the LCR to approach the sought circular track; and (ii) second, we deployed the FCR and updated the pose of the LCR by accounting for the contact actions exchanged by the CRs. The main idea was as follows: as the probe section nears the circular track, the contact actions can be described by a torque T(κ) that is only dependent on κ (and the above two-step approach applies), whereas the stiffness of the locked CR also decreases by increasing the span ℓ. For values of ℓ above a certain value, the probe section cannot accurately approach the sought track. We thus computed the deviation from the circular shape (both linear δ and angular δθ, refer to Fig 1D) and, once introduced an accuracy threshold ϵ = 5% on δ/ϕ and δθ, we determined ℓ = ℓ(κ_des_, ϕ, E, ν, ϕ_R_, d, ϵ). By symmetry, the two-step approach also applies by reversing the roles of the CRs (Fig 1D depicts an example configuration).

For completeness, we modeled the rods as simple Cosserat rods to enable large deformations [35] and we exploited the distal disk to couple the rods (similar to parallel continuum manipulators [36]). At each step, we determined the equilibrium pose by solving a minimization problem with Matlab (The Mathworks, Natick, MA, USA) based on the numerical integration of the underlying differential problem. Additional details are reported in S1 Text.

The main model results are depicted in the graph in Fig 1D. We considered κϕ = 1/5 based on the capabilities stated in [20] and the targeted curvature κϕ = 1/2 (for reference, the graph also shows the results for κϕ = 1, which was not addressed at this early stage). Based on the model results, we selected ℓ/ϕ = 1.5 for the probe span. As a model byproduct, we also obtained a reference value for the maximum rod-clamping force to be achieved with the shape-locker detailed design. By assuming a clamping element composed of aluminum, we targeted a clamping force approximately 10 N. Additional details are provided in S1 Text.

### Design and fabrication

**Fig 2:**
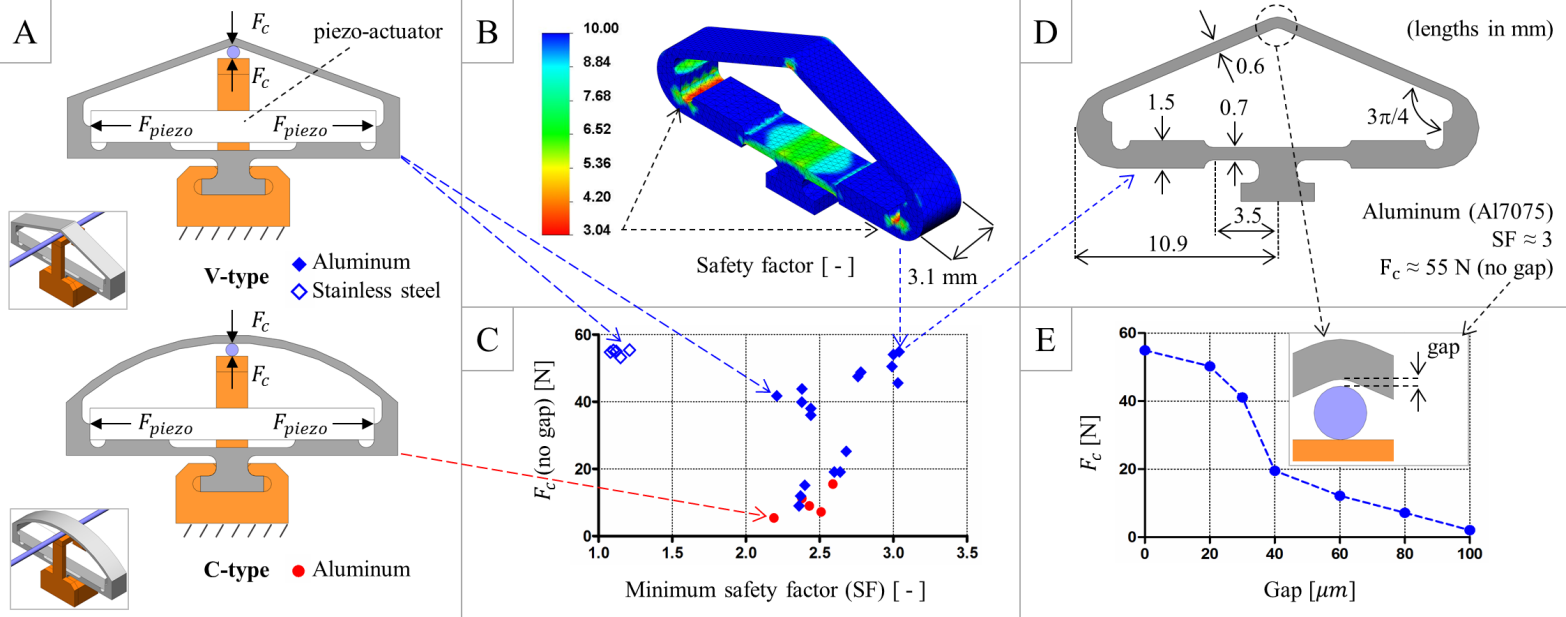
Piezo-clamp design optimization. (A) Prototypical solid models considered for design optimization. (B) Example results (material safety factor) obtained by FEA. (C) Optimization chart: we simultaneously maximized the rod-clamping force and the minimum safety factor of the piezo-clamp material. (D) Optimized profile of the piezo-clamp, which highlights the geometrical quantities that vary during the optimization process. (E) Sensitivity of the rod-clamping force (as obtained by FEA) to the gap between the rod and the piezo-clamp.

We implemented rod clamping using piezoelectric actuators to obtain relatively high forces (with small displacements) and reliability and short response times. We selected 3×2×18 mm P-882.51 piezo-stacks (PI GmbH, Karlsruhe, Germany), which feature a load-free expansion of 18 μm and a maximum blocking force of 210 N at 125 V. The size of the selected piezo-stacks was compatible with the assumed probe diameter. We designed the piezo-clamp (active clamping element): the solid models in Fig 2A, which were created with SolidWorks (Dassault Systèmes, Vélizy-Villacoublay, France), were optimized by finite-element analysis (FEA) using SolidWorks and Abaqus (Dassault Systèmes, Vélizy-Villacoublay, France). We simultaneously maximized the rod-clamping force and the material safety factor (Fig 2B and 2C) using the piezo-stack maximum blocking force as an input while considering the upper bound on the piezo-stack expansion. We selected the V-type piezo-clamp because it permits a more efficient transduction of the piezo-stack force to the clamping region, and we selected aluminum (Al7075) as the structural material because it yields larger safety factors compared with stainless steel. The optimized clamp profile (Fig 2D) features flexure-like sections in the lower part, which help convert the horizontal piezo-stack elongation into an effective vertical clutching on the rod: the simulation returned a 55 N clamping force when no gap exists between the rod and the piezo-clamp, with a minimum safety factor of 3. The clamping force was expected to overcome the targeted vale (10 N) for a maximum gap of 70 μm (Fig 2E). Considering the tolerances that are ordinarily achievable using current milling machines, we adopted the optimized profile to finalize the shape-locker design.

**Fig 3:**
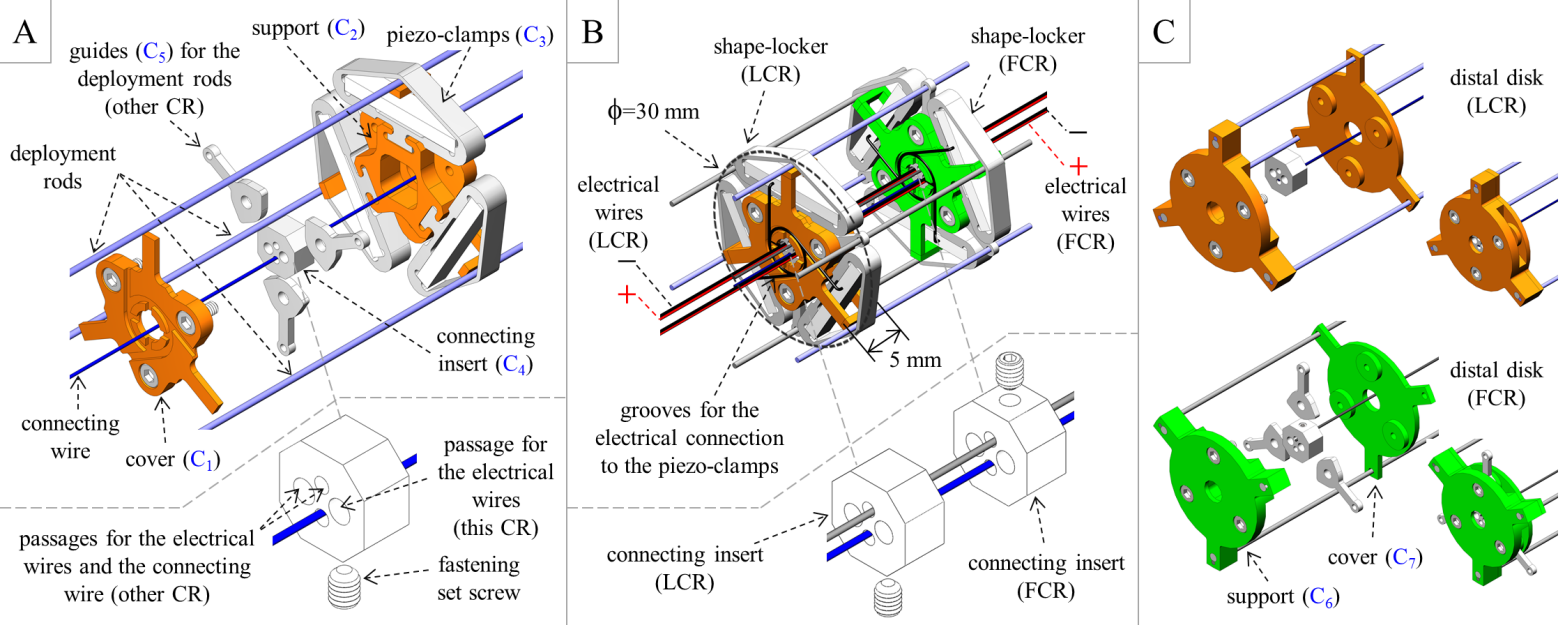
Design of shape-lockers and distal disks. (A) Shape-locker design (exploded view). (B) Assembly of two facing shape-lockers (the first shape-locker belongs to the leader, whereas the second shape-locker belongs to the follower), which highlights the π rad angular shift between them. (C) Distal disk design (exploded view and assembly). The seven different components needed to build the entire probe (apart from the commercial items) are labeled as C_1_ to C_7_.

The shape-locker design is shown in Fig 3A and 3B. For simplicity, we only considered one connecting wire (thus setting the angular shift between the CRs to π rad); we selected a 0.4 mm diameter NiTi rod (Euroflex, Pforzheim, Germany). We fastened the shape-locker to the connecting wire through an insert, which also featured the passages for the electrical wires that power the piezo-stacks. This insert and the guides for the deployment rods of “the other CR” were sandwiched between two structural elements, which are denoted as the cover and the support in Fig 3A, to inhibit rotations. Because the connecting wire was near the ideal probe centerline, its sliding through the insert of “the other CR” was expected to minimally affect probe deployment. Note that we only needed two electrical wires to power an entire CR (piezo-stacks in series). We also introduced some grooves on the cover and the support (those on the support are not visible in the figure) to prevent the electrical wires from invading the space between two adjacent shape-lockers (the space spanned by “the other CR”). The distal disks are shown in Fig 3C. We directly assembled the deployment rods to the disk support by interference (namely, through pins obtained from the same rods, which are not detailed in the figure), and we employed the previously mentioned insert and guides.

Once the disks are assembled, as shown in Fig 3, the probe features a strong symmetry. Fig 3 highlights how symmetry enables us to minimize the number of probe components: apart from the commercial items, we only need seven different components for the entire probe. These components are labeled as C_1_ to C_7_ in Fig 3, and the corresponding number of instances that are needed to assemble a probe with n active segments is reported in Table 1. Fig 3 demonstrates that symmetry also simplifies the assembly process: after tensioning the connecting wires, all shape-lockers are sequentially assembled by repeating the same operations (up to a π rad rotation) before piling the distal disk of the FCR (with its deployment rods). A single re-fixing is subsequently needed to finalize the assembly by piling the distal disk of the LCR (with its rods).

**Table 1:** Number of components for a probe with n active segments (*)

Component	Number of instances
C_1_: Shape-locker cover	2n
C_2_: Shape-locker support	2n
C_3_: Piezo-clamp	6n
C_4_: Connecting insert	2n+2
C_5_: Guides for the deployment rods	6n+3
C_6_: Distal disk support	2
C_7_: Distal disk cover	2

(*) Additional commercial items: 6+2 NiTi rods; 2n+2 set screws; 6n+3 screws; 6n piezo-stacks; 4 electrical wires (driving electronics excluded).

**Fig 4:**
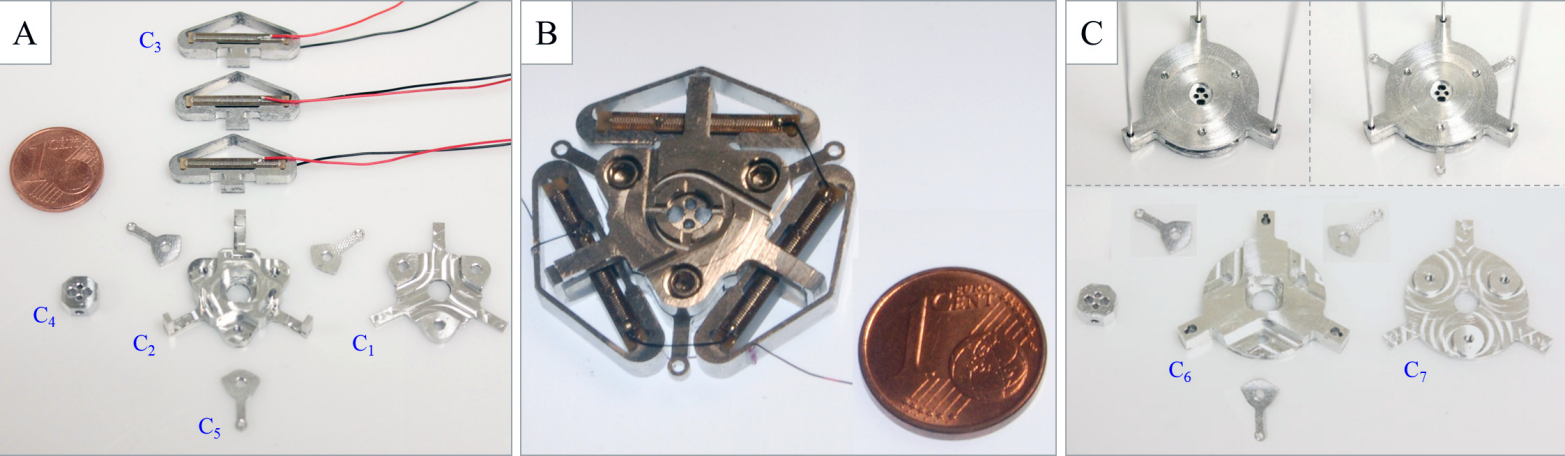
Fabricated shape-lockers and distal disks. (A) Fabricated shape-locker components, in which the piezo-clamps frame the piezo-stacks (as obtained from the commercial provider). (B) Shape-locker assembly, with the piezo-stacks properly connected in series. (C) Fabricated distal disk components and disks assembly. The seven different components required to build the entire probe (apart from the commercial items) are labeled as C_1_ to C_7_.

With regard to fabrication, we employed 3-axis CNC micro-milling to machine the probe components in Table 1. We selected the same material (Al7075) for each component for simplicity. Fig 4 shows the shape-lockers and the distal disks (the components and the assembly) and highlights the seven probe components that were developed in-house. In Fig 4A the piezo-stacks are framed in the piezo-clamps; they feature the electrical wires as soldered by the commercial provider. Fig 4B shows a shape-locker assembly in which the piezo-stacks are properly connected in series. As shown in Fig 4B, we did not use the grooves (yet the piezo-stack connection does not invade the workspace between two facing shape-lockers). We made the four electrical wires run externally (and close) to the probe for ease of assembly/testing. Fig 4C shows the only (negligible) difference between the two CRs, namely, the three guides on the distal disk of the FCR (also refer to Table 1). Note that the current size of the shape-lockers, including their thickness (refer to Fig 3B), was strictly dictated by the size of the commercial piezo-stacks.

**Fig 5:**
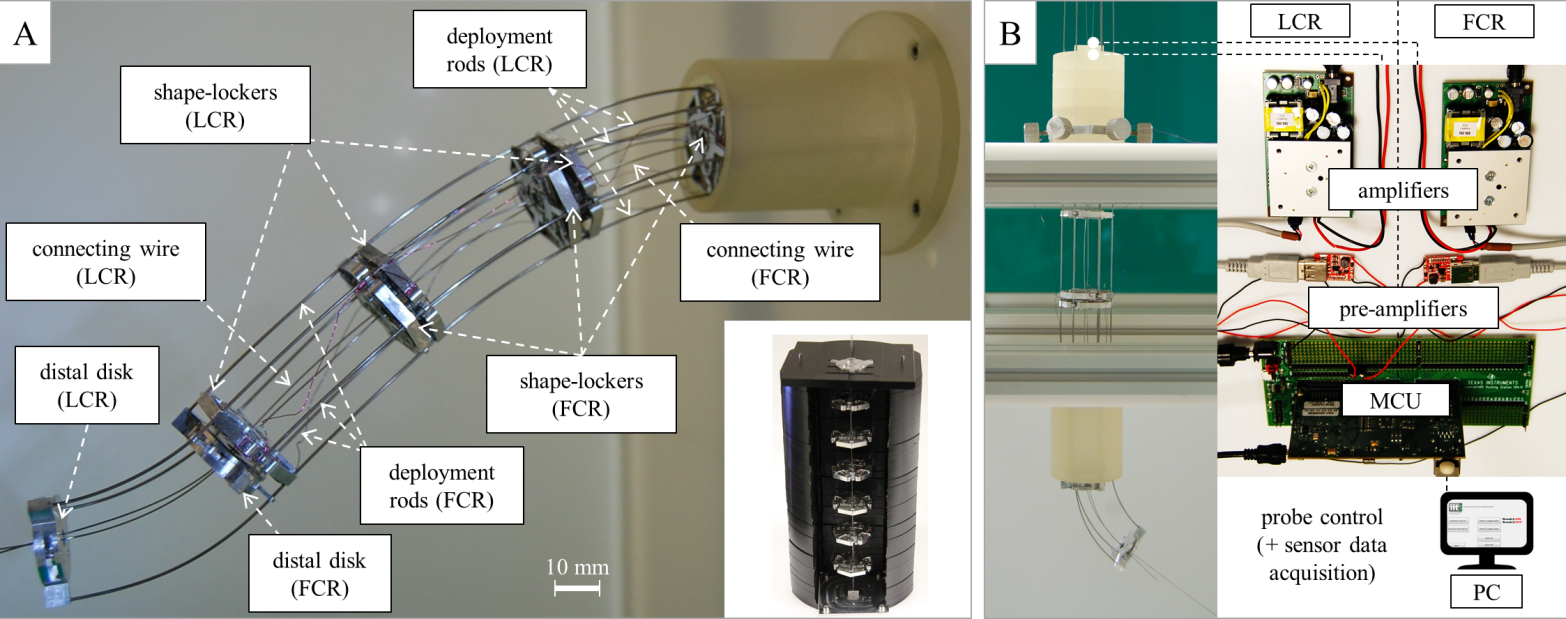
Probe prototype. (A) Probe prototype, as framed in the auxiliary support, and assembly stack (inset). (B) Driving electronics for the piezo-stacks, which are schematically connected to the control interface.

We fabricated a probe with n = 3 active segments. We inserted the prototype in an auxiliary support, which also features a flange (to encase the sections to be deployed), and a proximal guide with six clamping knobs to fasten the rods (in place of the actuators interface). To activate the piezo-stacks, we introduced a TI F25M36 MCU (Texas instrument, Dallas, TX, USA), which was controlled via a Qt-based user interface that runs on a common PC. To maximize clamping, we operated the piezo-stacks at 125 V and 200 mA. For each CR, we maintained this voltage using a Mini DC converter pre-amplifier (DROCK, Guangzhou, China) and an E835 OEM piezo amplifier (PI GmbH, Karlsruhe, Germany). Fig 5 shows the probe prototype, as framed in the auxiliary support (the flange and the knobs on the proximal guide are also visible) and deployed. The inset in Fig 5A details the auxiliary stack that is used to sequentially assemble the probe, as previously discussed. Fig 5B also illustrates the electronics that are employed to drive the piezo-stacks, which are schematically connected to the control interface.

## Experimental Assessment

We preliminarily measured the clamping force of a single shape-locker: we clamped the three rods through a suitable interface (with screws) and pulled this interface until slipping between the rods and the (activated) piezo-clamps was achieved. We acquired the pulling force at 62.5 Hz through a Nano17 force sensor (ATI Industrial Automation, Apex, NC, USA), which was linked to the previously mentioned interface through a gripper. We repeated the measurement six times. We achieved slipping when pulling the wires with 27.2±0.7 N. By assuming a static friction coefficient of approximately 0.45, as indicated in S1 Text (pertinent references are provided), the corresponding clamping force on a single rod was approximately 20 N. Thus, we obtained a proper margin with respect to the target value. Based on the FEA simulations in Fig 2E, this result reflected proper machining and fostered prototyping of the probe.

**Fig 6:**
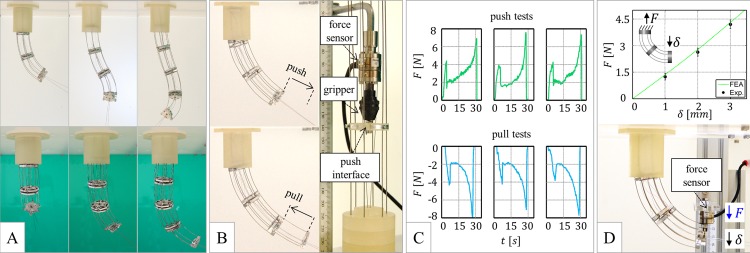
Probe experimental assessment: push/pull force and stiffness. (A) Examples of 3D poses obtained by differential rod pulling (top row: front views; bottom row: corresponding side views). (B) Probe configuration for the push/pull force tests, and set-up for the push tests. (C) Measured trends of the push/pull force versus time (three acquisitions). (D) Probe configuration for the stiffness tests (bottom), and relevant measures (top) compared with FEA simulations (graph inset).

We employed the previously mentioned sensor and clamping interface to assess the probe pushing force: we measured the force necessary to deploy the LCR for one span over the locked FCR by manually moving the LCR at nearly 1.5 mm s^-1^ (faster dynamics were not of interest at an early stage). We also considered the LCR reverse motion: by directly gripping and pulling the connecting wire (at the same speed), we measured the corresponding retraction force. We repeated both measurements six times. The probe achieved 3D poses by differential rod pulling (Fig 6A); the configuration that was considered for measuring the push/pull force is reported in Fig 6B, where the sensor and the auxiliary interface are also visible. Three examples of the trends of the measured force are shown in Fig 6C. We obtained repeatable results and similar results for deployment and retraction (which supports reversibility). We systematically measured a static friction threshold of approximately 4 N, after which the force settled at approximately 2 N before continuously increasing to nearly 8 N until the probe movement over the span was completed. This increasing trend can be attributed to the non-negligible thickness of the shape-lockers: the moving shape-lockers are steered when approaching the fixed shape-lockers and their thickness imposes a dynamic constraint (by flattening the passing-through rods, which can locally slide relative to the piezo-clamp in the unlocked configuration) that stiffens as the gap between the facing shape-lockers disappears. Potential effects, e.g., related to the probe configuration that was specifically considered for the measure, should be investigated. We measured an operating push/pull force in the range of approximately 4–8 N.

We also utilized the force sensor to measure the probe stiffness in a reference configuration. First, we deployed two probe sections (approximately over a quarter of circumference) and we locked both CRs. Second, we displaced the probe by pushing its tip with the force sensor, which was constrained to slide on a linear guide. We acquired the force that corresponds to selected displacements (1, 2 and 3 mm), and we repeated the measurement six times. The results of the stiffness test are shown in Fig 6D: the measured stiffness was approximately 1.5 N mm^-1^. To accurately interpret the experimental results, we also performed finite-element simulations: the experimental points matched the numerical results and highlighted that no slip occurred at the piezo-clamps (the simulations provided a slightly stiffer trend due to the ideal clamping assumed at the probe base).

**Fig 7:**
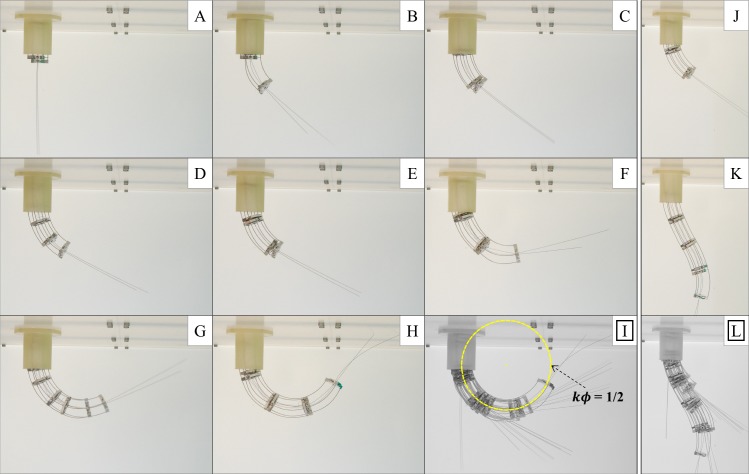
Probe experimental assessment: FTL deployment. (A-H) Probe deployment over a circular trajectory with a radius of curvature as low as twice the probe diameter. (I) Image overlay based on the sequence of images in A-H: the probe accurately achieves FTL deployment. (J-L) Probe deployment over a double curvature trajectory (example frames and image overlay): FTL deployment can be achieved, even if it is more challenging than deployment over a single curvature track. (The wires extending beyond the distal disk of the LCR are the continuation of the connecting wires; they were left after assembly just for ease of handling.)

Last, we also assessed the track-building capabilities: we deployed the probe over planar trajectories, with single and double curvature, by taking photographs. By fastening/releasing the clamping knobs, we alternately deployed the LCR and the FCR by manually pushing rod base clampers (with screws) constrained on auxiliary linear guides (aligned with the proximal guide), so as to mimic the linear actuation stage. We performed the single curvature tests by using a constant differential pushing for the LCR and we quantified the corresponding curvature since the first deployment step by standard image processing with Matlab (The Mathworks, Natick, MA, USA). For the double curvature tests we used a variable differential pushing for the LCR. At each step we pushed the FCR so as to catch up with the LCR. Track building was finally assessed through image overlays using Matlab. The probe achieved track-building with a radius of curvature as low as twice the probe diameter, as shown in Fig 7A–7I and outperformed state-of-the-art deployable tools [16,18,20]. These results fully supported the model-based approach that we proposed for design ice-breaking. Track-building was more challenging when pursuing double curvature trajectories, as shown in Fig 7J–7L. The obtained follow-the-leader performance is commensurate with the developmental stage. We achieved the design goal for this study. An example of deployment is shown in S1 Video, after the main contributions of this study are recalled.

## Discussion

Based on the obtained results, we successfully developed the first interlaced continuum robot and we fully achieved our goal by demonstrating FTL capabilities beyond the state of the art. Compared with the outstanding articulated device that was originally introduced in [12], our probe achieves higher curvature: we demonstrated κϕ = 0.5, while a maximum curvature κϕ<0.3 is stated [20]. More generally, our design enables us to modulate the deployment length with continuity (to the span value); therefore, it can easily conform to smooth trajectories. Additional considerations could be drawn based on the FTL capabilities of the articulated system, which are not quantitatively assessed in the literature, to the best of our knowledge. Furthermore, our probe achieves curvatures that are one order of magnitude higher than the curvatures of concentric tube robots [18]. More remarkably, compared with the concentric tube robot discussed in [18], our design achieves FTL deployment without *a priori* limitations to very specific trajectories, thus intrinsically spanning a larger workspace. For the devised interlaced robot, the deployment track can be modulated at runtime, whereas it is *a priori* hard-coded for concentric tube robots. This aspect expands the application potential of our design to scenarios that benefit from a dynamical mapping of the workspace (including the target and the possible obstacles to avoid), as obtained by e.g., visual inspection.

We are aware of the fact that the current prototype has many limitations. First, the size of the piezo-stacks currently sets the probe diameter and the thickness of the disks, which affects the track-building capability and the operating force, as previously mentioned. Although probe miniaturization may be desirable for accessing narrower sites, it should be negotiated with the probe stiffness (miniature tools usually confine bending to the tip region [37]). An adverse scaling effect is encountered when the number of active segments (and the probe length) with a fixed diameter increases due to an increase in frictional effects and a reduction in stiffness. For instance, our prototype exhibits deformations of a few mm when loaded with e.g., 5 N; thus, its potential payload is similar to the payload considered in [12]. It is stiffer than the continuum device in [26] and substantially stiffer than concentric tube robots [38]. The probe stiffness depends on its pose and on the direction of the applied load. We showed that changing the differential deployment of the rods (as necessary to build double curvature tracks) directly opposes shape-keeping, and this effect can be pronounced based on stiffness of the specific probe pose. However, we did not explore this aspect, which must be handled by any applied embodiments of the proposed design (stiffness scaling can be better addressed when focusing on a specific application). A model-based approach, such as the approach proposed in S1 Text, may be initially invoked; however, it should be complemented with a model for the shape-locking actions. Indeed, the piezoelectric technology may become ineffective below a certain size, and competing options (based on shape-memory alloys [39]) should be investigated. Although these investigations are beyond the scope of this paper, they are enabled by the fact that the proposed probe relies on scalable technologies.

Moreover, starting from scratch the design of a completely new device, we could not reach a complete system: we demonstrated a partially complete system due to the lack of an actuation unit. Based on the measured operating forces (approximately 4–8 N at 1.5 mm s^-1^), common linear stages, which also feature power margins for faster probe operations, can be employed [12,28–30]. We did not address the impact of pulling mechanisms, which are necessary for the retraction of the connecting wires, on the actuation requirements. The integration of motorized spools should not perturb the classical linear stage arrangement [12,28–30] thanks to the fact that the connecting wires run close to the probe centerline and they need not be driven on a straight path. The envisaged off-board actuation helps contain the system complexity (and provides some safety margins). However, the actuators should be tailored based on a specific application. Consistently, we did not study probe control, which could be tackled starting from an approach similar to the one in [26] (at least partially, since the probe deployment adds to the complexity of the problem). Because of these limitations, we could not compare the achieved tracks with pre-planned trajectories (yet we could demonstrate track-building through image processing).

Finally, we did not considered further issues, such as the integration of working channels (according to the concept) and a probe cover.

In the next developmental step, electrical wiring should be improved by exploiting available passages/grooves and should be made fail-safe by introducing some redundancy. Moreover, a refined modeling framework, such as the framework presented in [40–41], should be introduced. This framework could effectively address probe scaling (by incorporating frictional effects, difficult to characterize) and stability (the locked CR helps stabilize the rods of the unlocked CR; however, adverse effects are expected when increasing the rod length). Furthermore, the probe should be endowed with some sensing capabilities to estimate its actual pose, as functional to control (refer to e.g. [42–44]). The previously described limitations, which must be addressed in future studies, do not seem to weaken the proposed design and the results of this study. However, application-specific constraints may facilitate the redesign of some probe components.

## Concluding Remarks

We conceived the first interlaced continuum robot, which paves the way for a new class of continuum systems [27]. We developed an interlaced configuration by pursuing a conceptual approach: we questioned the possibility of solving the FTL deployment problem by harnessing symmetry in the design process. We addressed the possibility for a continuum probe to follow the leader through cavities, without the aid of external supports and without relying on special operating conditions. This intrinsic capability is suitable for high-impact applications such as visual inspection and medical robotics, in which contactless deployment contributes added value (and may enable new procedures). This study discusses the initial and extensive development of the probe: model-based conceptual design (grounded on mathematical modeling), detailed design and prototyping, and experimental assessment.

A distinctive aspect of our conceptual approach must be remarked. Regardless of application-specific constraints, once two CRs with controllable stiffness were assumed to address physical track-building by an alternating method, the interlaced configuration stemmed from symmetry. The numerous advantages due to symmetry are evident; it enables the following actions: (i) to minimize the probe cross-section for a given bending stiffness of each CR; (ii) to reduce, in general, the distance between the bending neutral axes that are ideally associated with the CRs to enhance shape-keeping while the unlocked CR travels onto the locked CR; (iii) to remarkably address the model-based span determination by considering a single probe section, which renders the quantitative approach to design viable; (iv) to minimize the number of probe components; (v) to sequentially assemble the probe using a single auxiliary stack; (vi) to ideally simplify control using corresponding control laws for the CRs, both in the deployment phase and the retraction phase (also based on minimal electrical wiring for shape-locking). We believe that we could not have achieved a completely new probe without the previously mentioned conceptual approach to design, which can be employed when considering interlaced systems with stronger symmetry (for instance, when using more rods).

Despite the limitations that are inherently associated with its original character, the main contribution of our study is three-fold: a prototypical approach to the design of interlaced continuum systems; the first embodiment of an interlaced continuum system; and a continuum probe that is intrinsically able to follow the leader.

## Supporting Information

S1 Text Model-based design ice-breaking. We developed a mathematical model to determine the probe span based on a target curvature for the trajectory to be achieved by track-building. The model, which is primarily based on the equilibrium of simple Cosserat rods, also provided a reference value for the rod-clamping force. We solved the underlying differential problem by introducing a suitable optimization procedure.
(PDF)

S1 Video The first interlaced continuum robot, devised to intrinsically follow the leader. This video introduces the interlaced robot concept, recalls the entire development of the probe (concept, design and prototyping, and experimental assessment), and demonstrates a follow-the-leader deployment.
(MP4)
