# Supplementary material for: The First Interlaced Continuum Robot, Devised to Intrinsically Follow the Leader
Source: PLoS One. 2016 Feb 25;11(2):e0150278. doi: 10.1371/journal.pone.0150278 (PMC4767230; doi:10.1371/journal.pone.0150278)
Supplement: S1 Text — We developed a mathematical model to determine the probe span based on a target curvature for the trajectory to be achieved by track-building. The model, which is primarily based on the equilibrium of simple Cosserat rods, also provided a reference value for the rod-clamping force. We solved the underlying differential problem by introducing a suitable optimization procedure. (PDF) [file pone.0150278.s001.pdf]

The first interlaced continuum robot, devised to intrinsically follow the leader

B. Kang, R. Kojcev, E. Sinibaldi (edoardo.sinibaldi@iit.it)

Supporting Information S1: Model-based design ice-breaking

[Framework and main aim of the model]

Probe design was initialized by choosing the diameter ϕ (characteristic probe size, also shown in Figure 1A) and the deployment rods. In particular, we firstly chose $\phi = 30$ mm for ease of development, namely to address the detailed design of miniaturized shape-lockers while not introducing technological challenges incommensurate with the current developmental stage. Then, in order to confer enhanced elasticity to the probe, we selected commercially available superelastic NiTi rods (Euroflex, Pforzheim, Germany) with Young modulus $E \cong 58$ GPa and Poisson ratio $\nu \cong 0.33$ [1-3]. In particular, with reference to Figure 1B, we chose $\phi_R = 0.8$ mm (rod diameter) and $d = 14$ mm (distance of the rod center from the center of the shape-locker; it is the same for the distal disk). We deliberately selected a rather extremal value for d in order to maximize the probe bending stiffness in the locked configuration (by maximizing the area moment of inertia of the rods, with reference to classical beam theory [4]).

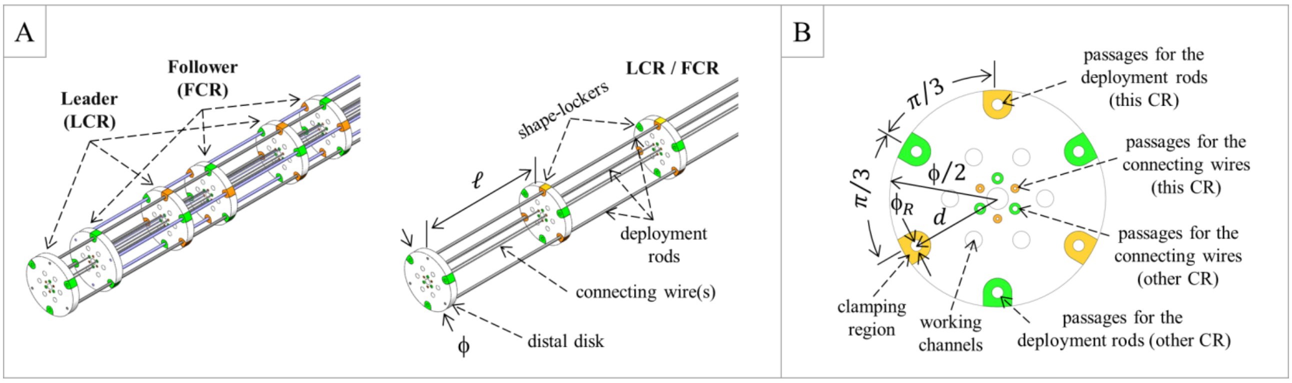

Figure 1. Probe concept. (A) Schematic of the probe consisting of two interlaced continuum robots (CRs); the probe diameter ϕ and the span ℓ are also indicated, together with the main components of each robot. (B) Shape-locker concept highlighting the strong symmetry that underpins the interlaced configuration: each shape-locker must enable the simultaneous operation of both CRs. (This figure replicates Figures 1A-B of the main text, for ease of presentation.)

We then introduced a mathematical model in order to obtain a design value for the probe span ℓ (indicated in Figure 1A). In more detail, we studied the statics of an idealized probe section to be deployed so as to build a circular track portion with curvature κ (we considered a quasi-static deployment, as most likely for common applications). For any fixed value of κ, the model aimed at determining the span $\hat{\ell} = \hat{\ell}(\kappa)$ that allows the probe section to build the sought track portion, within a chosen relative tolerance ϵ. Such a tolerance is properly defined in the sequel, yet let us anticipate that we adopted $\epsilon = 5$ %, so that all the fixed working parameters (namely ϕ, E, ν, ϕ_R, d and ϵ) are now stated.

Therefore, formally speaking, we computed

$$\hat{\ell} = \hat{\ell}(\kappa; \phi, E, \nu, \phi_R, d, \epsilon), \quad (1)$$

for selected values of κ. Then, we targeted $\kappa_{des} = (2\phi)^{-1}$ as design curvature: we aimed at achieving a radius of curvature as low as twice the probe diameter during the deployment. This is very challenging; indeed, it is beyond the capabilities of relevant state-of-the-art systems [5-7]. Hence, we used the developed model for determining the design value of the probe span, as follows:

$$\ell \cong \hat{\ell}((2\phi)^{-1}; \phi, E, \nu, \phi_R, d, \epsilon). \quad (2)$$

In the sequel we detail the procedure that we used for obtaining $\hat{\ell}$ as in Eq. 1, and we show exemplificative results including those providing ℓ according to Eq. 2.

Let us finally remark that the proposed model is not tied to a specific characteristic size of the probe: the working parameters can be varied in light of model objectives and constraints (the parameters set is a matter of design). Furthermore, let us anticipate that, as a byproduct of the model, we also obtained a reference value for the rod-clamping force to be achieved through the shape-lockers. This is briefly reported at the end of the present document.

[Modeling approach and working assumptions]

We addressed the following basic problem: how to build a circular track portion with curvature κ by deploying an idealized probe section like the one sketched in Figure 2. The section at hand bends in a plane that contains two of the six deployment rods, for simplicity, and its shape is defined starting from the centerline (dashed in the figure), namely a circular arc with curvature κ and length ℓ. (With minor abuse of notation, we use the same symbol for the centerline length and for the design value of the probe span.)

In particular, the deployment rods are defined through circular arcs that subtend the same angle $\theta = \kappa\ell$ as the centerline. Moreover, the proximal/distal disks (introduced to lock the LCR shape) have negligible thickness and they are perpendicular to the centerline. Furthermore, we neglect the connecting wires because their contribution to the probe bending stiffness is negligible compared to the one of the rods (by construction), and therefore they are not relevant for the subsequent equilibrium problem. Finally, based on the relative position of the continuum robots (CRs) with respect to the bending plane, the shortest arc either belongs to the leader (LCR), as in Figure 2A, or to the follower (FCR), as in Figure 2B. The former configuration is labelled "FP" and the latter "UP", for ease of presentation.

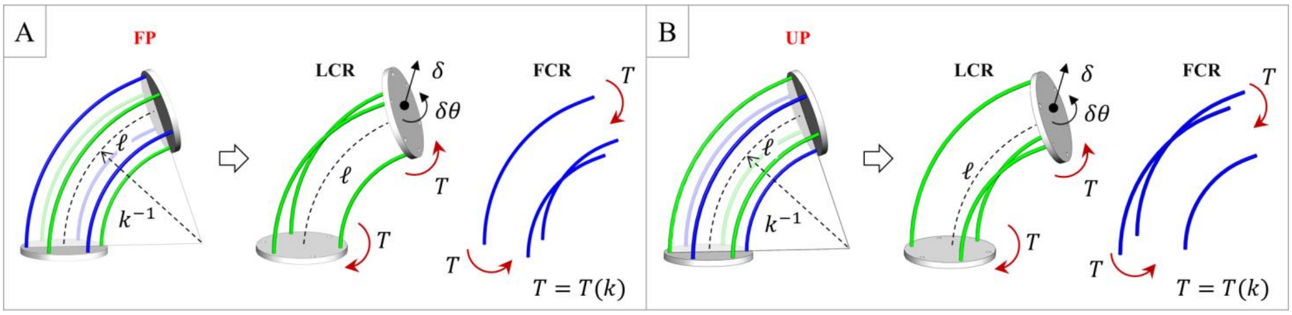

Figure 2. Schematic of an idealized probe section that builds a circular track portion. The probe features a span ℓ, while κ denotes the track curvature. The shortest circular arc either belongs (A) to the leader or (B) to the follower. Track building was addressed by a two-step simplified approach: we firstly determined the load-free pose of the leader (locked, green in the figure) that approximates the circular shape. We then applied the torque T associated with the bended rods of the follower (unlocked, blue in the figure), and we studied the resulting deviations (linear δ and angular $\delta\theta$) from the circular shape. For a chosen κ we obtained the maximum ℓ that permits to approximate the circular shape within a chosen accuracy. Span determination was based on this approach, which holds when also reversing the roles of the CRs, by symmetry. (The B-subfigure is included in Figure 1D of the main text, for ease of presentation.)

We tackled the track-building problem by a simplified approach, namely by studying two (static) equilibrium problems associated with the following deployment steps:

- [*Step#1: The unlocked LCR is firstly deployed so as to finally approach the pursued circular shape; then, its shape is locked. Upon successful completion of this step, the equilibrium configuration of the LCR suitably approximates the sought circular track.*] We firstly determined the load-free equilibrium pose of the LCR, by minimizing the distance δ of the distal disk center from the endpoint of the pursued circular centerline, and the angular deviation $\delta\theta$ from the angle θ that defines the pursued pose of the distal disk. We determined the LCR rod lengths as part of the solution (as for an inverse-kinematics problem);
- [*Step#2: The unlocked FCR is then deployed over the locked LCR. Upon successful completion of this step (i.e. provided that the LCR section is stiff enough), the resulting probe section still suitably approximates the pursed circular track.*] We then studied the equilibrium pose of the LCR (with the rod lengths determined at Step#1) by accounting for the contact with the FCR. Also in this case we minimized δ and $\delta\theta$, and we regarded the resulting pose as a suitable approximation of the pursued track when

$$\max\left(\frac{\delta}{\phi}, \delta\theta\right) \leq \epsilon, \tag{3}$$

where ϵ denotes the aforementioned non-dimensional tolerance.

In more detail, at Step#2 we assumed that each of the FCR rods only acted on the LCR through a bending torque $\mathrm{T} = \mathrm{T}(\kappa)$ depending on the assumed curvature. This is consistent with the constant-curvature assumption of the rods (also for large displacements/deformations [8]), as far as the resulting equilibrium pose of the probe section is close enough to the pursued circular shape. In other words:

- When the condition in Eq. 3 holds: the locked (and loaded) LCR section is reasonably close to the pursued circular shape; the FCR rods (as guided by the LCR) are correspondingly close to the associated circular arcs of the circular shape; the mechanical actions exchanged by LCR and FCR can

be reasonably described through pure bending torques, as discussed above. In this case, therefore, the model solution is physically-representative (for the statics at hand), and the considered section manages to build the track;

- When the condition in Eq. 3 is violated, the considered section cannot be deployed so as to build the pursued track. In this case, the model solution at Step#2 does not accurately describe the actual statics, in particular because the FCR rods substantially differ from the assumed circular arcs, and therefore the mechanical actions exchanged by LCR and FCR are no longer described by pure bending torques. Indeed, the actual pose of the probe section must be determined by solving the coupled problem (LCR and FCR): the two-step approach is no longer suitable. However, we are not interested in static solutions that violate Eq. 3: we are only interested in determining the upper value of ℓ for which Eq. 3 holds. Hence, the proposed two-step approach is commensurate with the model objectives, and can be effectively used for determining the probe span;

Indeed, the main idea behind the proposed two-step approach is that deviations from the circular shape can be contained, provided that ℓ is low-enough. In fact, for any fixed values of κ the torque applied by the unlocked CR only depends on κ (as far as the rods are circular), yet the stiffness of the locked CR decreases when increasing ℓ.

In general, one might get close to the circular shape at the end of Step#1, yet by approaching unstable configurations of the rods. In that condition, however, the subsequent loading at Step#2 most likely causes the loaded LCR to substantially depart from the sought track. Hence, the proposed modeling approach also detects unstable probe configurations, as exemplified by the results below.

Furthermore, let us remark that, once determined the probe span as discussed above, the conditions for proper track-building are expected to be met at the subsequent deployment step as well, thanks to the symmetry between LCR and FCR in our interlaced concept. Indeed, when building the subsequent track portion, the (unlocked) LCR can properly catch up with the (locked) FCR over the previously-built track portion, by symmetry. Of course, we are simplifying the scenario; for instance, we are not considering the stability of the cumulatively-built track, which can be perturbed during the deployment. However, the way we address track-building by considering a single probe section should be noticed: it is intrinsically based on the symmetry underpinning the interlaced concept, according to which LCR and FCR essentially play identical roles to implement a truly alternating method [9].

Finally, we supposed the deployment rods to be elastic (we assumed the same elastic and cross-sectional properties for all the rods), and we described them as simple Cosserat rods to allow for large displacements/deformations [8]. Moreover, we exploited the momentum balance of the distal disk to couple the mechanical actions of the rods (and thus the CRs at Step#2). Furthermore, we used the pose of the disks (both the distal and the proximal one) to introduce proper boundary conditions at the rods endpoints (ideal clamping assumption, like in [10]). In addition, we neglected gravity, to simplify the derivation and because it is expected to play a minor role for many probe poses (simple estimates support this point, see e.g. [11]). Let us also remark that we deliberately introduced a small set of model parameters in order to contain the uncertainties, and to obtain results effectively usable for probe development.

[Model formulation and solution strategy]

The shape of the i-th rod ($i = 1,2,3$; subscripts are hereafter understood) can be obtained by integrating the following ordinary differential equations [8]:

$$p_i' = R_i v_i, \tag{4}$$

$$R_i' = R_i (u_i)^\wedge, \tag{5}$$

$$n_i' = 0, \tag{6}$$

$$m_i' = n_i \times (R_i v_i), \tag{7}$$

to be complemented with proper boundary conditions. Symbols $p_i \in \mathbb{R}^3$ and $R_i \in SO(3)$ respectively denote the rod position and material orientation, which provide a body (i.e. material-attached) frame for each value of the arc length $s_i \in [0, \ell_i]$ (ℓ_i is the rod length). Furthermore, $n_i \in \mathbb{R}^3$ and $m_i \in \mathbb{R}^3$ indicate the internal force and torque, respectively. Moreover, $v_i \in \mathbb{R}^3$ and $u_i \in \mathbb{R}^3$ denote kinematic variables, expressed in the body frame, respectively associated with the linear and the angular rate of change of the body frame itself. In particular, v_i is related to extension/shear, while u_i is related to bending/torsion. In addition, the prime operator represents differentiation with respect to the arc length, while $(\cdot)^\wedge$ (hat operator) denotes the classical mapping from $\mathbb{R}^3$ to $so(3)$, which is the Lie algebra of $SO(3)$. Let us observe that we expressly neglect body forces and torques in the rod momentum balances (Eq. 6-7), consistently with the considered loading conditions. Furthermore, from Eq. 6 we immediately obtain that n_i is constant along the i-th rod, so that we can omit the formal dependence on the corresponding arc length (besides neglecting Eq. 6 itself).

Then, for an elastic rod the kinematic variables are linked to the internal force and torque by the following constitutive relations (that provide the closure for Eq. 4-7):

$$n_i = R_i K^{se} (v_i - v_i^*), \tag{8}$$

$$m_i = R_i K^{bt} (u_i - u_i^*), \tag{9}$$

where the starred quantities refer to the stress-free reference configuration of the rods. Moreover, $K^{se} = diag(AG, AG, AE)$ and $K^{bt} = diag(EI, EI, GJ)$ are stiffness matrices, where A, I and J respectively denote the area, the area moment of inertia and the polar moment of inertia of the cross-section, and $G = E/\big(2(1+\nu)\big)$ is the shear modulus. For the sake of definiteness, let us introduce a Cartesian frame such that the initially straight LCR rods (load-free and stress-free) are aligned with the z–axis (with the arc length increasing with z). Then, $v_i^* = [0,0,1]^T$ and $u_i^* = [0,0,0]^T$.

Let us now consider the LCR distal disk; its momentum balances read:

$$\sum_{i=1}^{3} n_i = 0, \tag{10}$$

$$\sum_{i=1}^{3}\left(p_i(\ell_i) \times n_i + m_i(\ell_i)\right) - m_f = 0, \tag{11}$$

where m_f denotes the torque due to contact with the FCR rods. Based on the two-step strategy introduced above, m_f is null at Step#1, while it has a known expression at Step#2 (reported below). It should be noticed that Eq. 10-11 provide a set of boundary conditions for the rods, which couple the internal forces and torques at the endpoints (the reference point for the angular momentum being immaterial).

Let us then consider the geometric boundary conditions for the rods. To the purpose, let r_i denote the relative position of the i-th rod's endpoint with respect to the center of a disk (either proximal or distal, being the same), as represented in the disk-attached frame. Moreover, let R_{dd} indicate the rotation of the distal disk, and p_{dd} the position of its center; furthermore, let R_{pd} and p_{pd} represent the corresponding quantities for the proximal disk. Then, the following compatibility conditions straightforwardly stem from the assumed perfect clamping:

$$p_{pd} + R_{pd}\, r_i - p_i(0) = 0, \tag{12}$$

$$\left(log\left(R_i^T(0)\ R_{pd}\right)\right)^{\vee} = 0. \tag{13}$$

$$p_{dd} + R_{dd}\, r_i - p_i(\ell_i) = 0, \tag{14}$$

$$\left(log\left(R_i^T(\ell_i)\ R_{dd}\right)\right)^{\vee} = 0. \tag{15}$$

In particular, Eq. 13 and Eq. 15 state that the rod orientation at the corresponding endpoints is the same as the one of the disks (the matrix logarithm and the $(\cdot)^{\vee}$ operator, which is the inverse of $(\cdot)^{\wedge}$, permit to use a compact notation, as e.g. in [10]).

We are now in a position to simplify the formulation, by exploiting the assumed planar deformation of the idealized probe section. To the purpose, let us assume that each rod deforms on a plane parallel to the xz – plane. Moreover, two of the three rods, say the ones with subscript 2 and 3, undergo the same deformation, thanks to the symmetry with respect to the $y = 0$ bending plane. Hence:

- We only solved Eq. 4 for the components x_i and z_i, with $i = 1,2$ (four equations). Moreover, we recast Eq. 5 in terms of a rotation angle α_i around the y-axis (two equations). Furthermore, given the internal force components n_{x1} and n_{z1} in rod#1, Eq. 10 immediately provides $n_{x2} = n_{x3} = -n_{x1}/2$ and $n_{z2} = n_{z3} = -n_{z1}/2$. Hence, we treated n_{x1} and n_{z1} as two unknown constants, tentatively assigned to integrate the differential problem yet to be determined as part of the solution process. In addition, Eq. 7 only provides a non-null component along the y –axis, so that we considered m_{yi} (two equations); similarly, also m_f appeared through the y –component only. Moreover, we consistently considered the relevant components of the closure relations, i.e. Eq. 8-9;

- As regards the proximal boundary conditions, we directly imposed the ones regarding x_i, z_i and α_i (six values), as provided by Eq. 12-13. Moreover, we treated the proximal values of m_y, say m_{y1}^0 and m_{y2}^0 as two additional unknown constants, tentatively assigned to integrate the differential problem yet to be determined as part of the solution process;
- At the distal end, based on Eq. 11 and Eq. 14-15, we defined the following residuals (let us omit the arc length specification for conciseness):

$$\rho_m \equiv m_1 + 2\, m_2 + n_{x1}(z_1 - z_2) + n_{z1}(x_2 - x_1) - m_f\ , \tag{16}$$

$$\rho_{xi} \equiv x_{dd} + [R_{dd}\, r_i]_x - x_i, \tag{17}$$

$$\rho_{zi} \equiv z_{dd} + [R_{dd}\, r_i]_z - z_i, \tag{18}$$

$$\rho_{\alpha i} \equiv \alpha_{dd} - \alpha_i, \tag{19}$$

to be minimized as part of the solution process (see below). The symbols $[\cdot]_x$ and $[\cdot]_z$ in Eq. 17-18 denote the projection on the corresponding coordinate axis, while x_{dd}, z_{dd} and α_{dd} clearly represent the position and the orientation of the distal disk. For convenience, however, we defined the latter quantities with reference to the pursued circular shape (see Figure 2). In particular, once introduced the coordinates (x_{cl}, z_{cl}) of the distal endpoint of the centerline, and by recalling the angle θ, we introduced the following deviations:

$$\delta_x \equiv x_{dd} - x_{cl}\ , \tag{20}$$

$$\delta_z \equiv z_{dd} - z_{cl}\ , \tag{21}$$

$$\delta\theta \equiv \alpha_{dd} - \theta\ , \tag{22}$$

to be determined through the minimization process (see below). The expressions of x_{cl} and z_{cl} in terms of ℓ and κ can be easily derived (see e.g. [12]); they are omitted for brevity.

In light of the above formulation, let us finally detail the solution strategy:

- [*Step#1*] We determined the LCR equilibrium pose by minimizing the seven residuals defined in Eq. 16-19; in particular, we chose their sum as cost function. To the purpose, we selected the following seven optimization variables: n_{x1}, n_{z1}, m_{y1}^0, m_{y2}^0, ℓ_1, ℓ_2 and $\delta\theta$;
- [*Step#2*] We performed the same minimization as for Step#1, yet with the following optimization variables: n_{x1}, n_{z1}, m_{y1}^0, m_{y2}^0, δ_x, δ_z and $\delta\theta$ (in particular, we adopted the rod lengths obtained at the previous step).

In more detail:

- We did not allow for linear deviations of distal disk position at Step#1 in order to approach the sought circular shape as closely as possible (i.e. consistently with the fixed model parameters). Conversely, at Step#2 we expressly introduced δ_x and δ_z, not to introduce unphysical constraints on the solution. Indeed, from Step#2 we obtained the values of $\delta\theta$ and $\delta \equiv (\delta_x^2 + \delta_z^2)^{1/2}$ necessary for assessing track-building capabilities according to Eq. 3;
- At Step#2 we assumed m_f to be given by the following expression [8]:

$$m_f \equiv EI\,\theta\, \sum_{j=1}^{3} \lambda_j^{-1}\,, \qquad (23)$$

  where λ_j is the length of the j-th FCR rod ($j = 1,2,3$) in the assumed circular shape (see Figure 2), so that $\theta\lambda_j^{-1}$ represents its curvature. For brevity, we omit the expression of λ_j (it can be easily obtained, see e.g. [12]), as well as the simplification of Eq. 23 enabled by symmetry;
- Once recast the problem in non-dimensional form, we numerically integrated the Cosserat differential equations in order to evaluate the cost function. To the purpose, we exploited a classical Dormand–Prince scheme available in Matlab (The Mathworks, Natick, MA, USA), with a tight relative tolerance (10^{-8}). Cost minimization was performed through Matlab as well, in particular by exploiting a quasi–Newton method (we assumed the optimization landscape to be locally smooth);
- For any fixed values of κ we solved a sequence of equilibrium problems, by increasing the centerline length. This way, we could exploit a previously obtained solution to guess the subsequent one (both at Step#1 and Step#2), with clear benefits on convergence and computational cost. This strategy also benefited from the fact that we formulated some of the residuals in terms of variations: as far as the probe achieves track-building, the deviations from the circular shape are small and the algorithm can easily converge by initially assuming null deviations. In addition, we could easily detect unstable rod configurations: stable configuration were incrementally explored up to e.g. a bifurcation (after which the solution was no longer of interest, as discussed above);
- In summary, once fixed κ, we obtained $\delta\theta$ and δ for increasing values of the centerline length, and we determined the limit value $\hat{\ell}(\kappa)$ based on the condition in Eq. 3. Finally, we obtained the design value for the probe span by specifically considering $\kappa_{des} = (2\phi)^{-1}$, i.e. by picking $\hat{\ell}((2\phi)^{-1})$.

[Model results]

Exemplificative results obtained through the proposed modeling strategy are reported in Figure 3 and Figure 4.

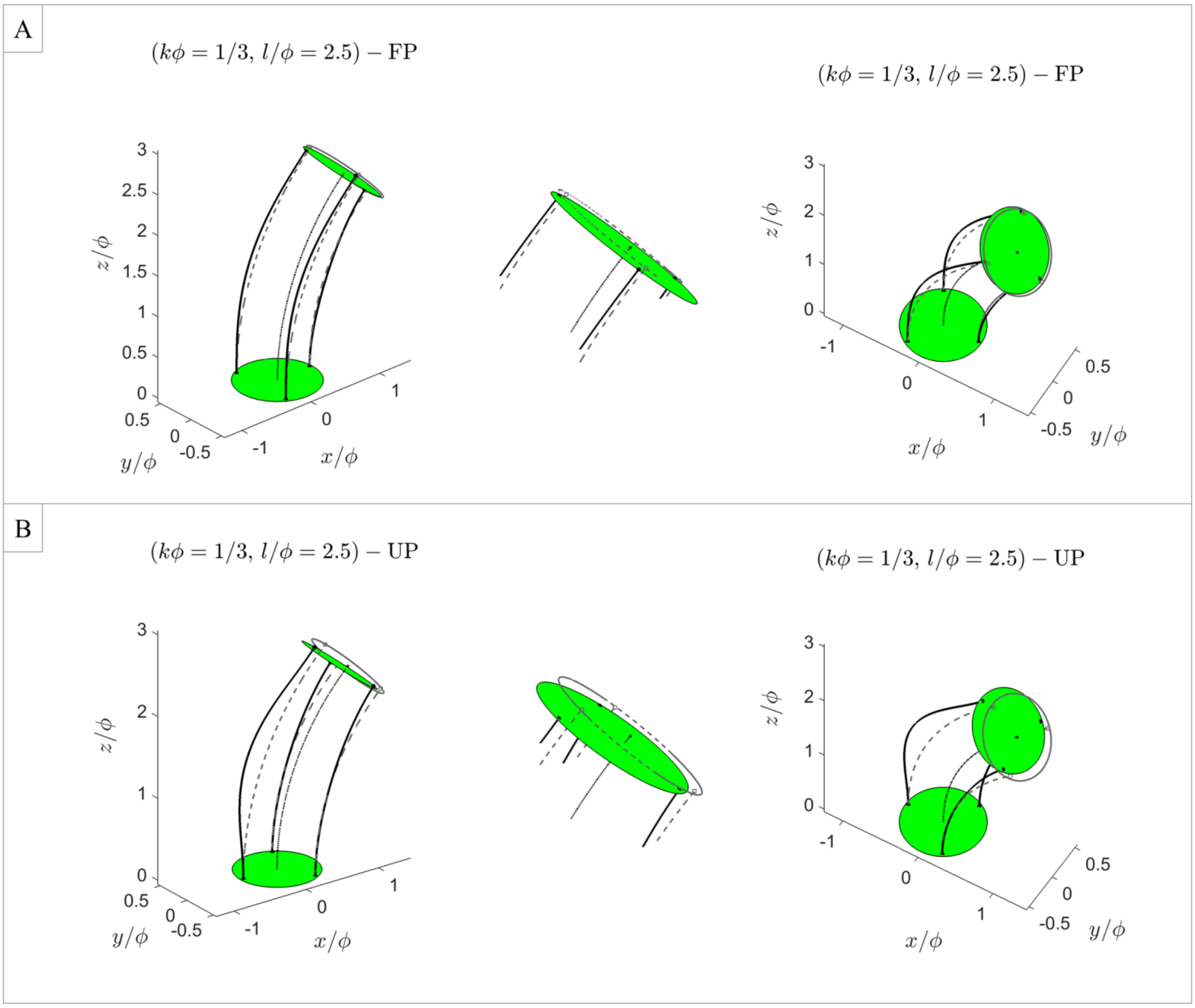

Figure 3. Equilibrium configuration of the idealized probe section, for $\kappa\phi = 1/3$ and $\ell/\phi = 2.5$. (A) LCR with the FP rod configuration. (B) LCR with the UP rod configuration. Each subfigure features two views (left and right) of the equilibrium pose, and a detail (middle) of the distal disk. The pursued circular track is also sketched (dashed curves denoting the arcs, the centerline and the distal disk); the FCR rods are not shown for ease of representation. Mechanical instability prevents the probe section in (B) from accurately building the pursued track.

In particular, in Figure 3 the FP configuration permits to approximate the pursued circular shape, while the UP configuration exhibits rod instabilities and substantially departs from the sought circular track. This is consistent with physical intuition: it is more challenging to achieve a chosen curvature by only pushing with one rod (instead of two). Indeed, we introduced the labels FP and UP to remind of favorable and unfavorable pushing, respectively, for the LCR rods. Yet the careful reader will spot that the strong symmetry underlying our interlaced probe concept should not introduce any bias that favors one rod configuration with respect to the other. And this is indeed the case, since at each deployment move the FP configuration of the LCR

corresponds to the UP configuration of the FCR, and vice-versa. Therefore, from Figure 3 we obtain a unique information: we conclude that the span $\ell = 2.5\,\phi$ is too long for accurately building a track with radius of curvature equal to 3ϕ.

Figure 4 then exemplifies the instability faced when crossing the $\hat{\ell}$ threshold, in particular for $\kappa\phi = 1/2$ (and by considering the UP rod configuration). In more detail, Figure 4 shows that accurate track-building can be achieved by choosing $\ell = \phi$, while $\ell = 2\phi$ leads to instability.

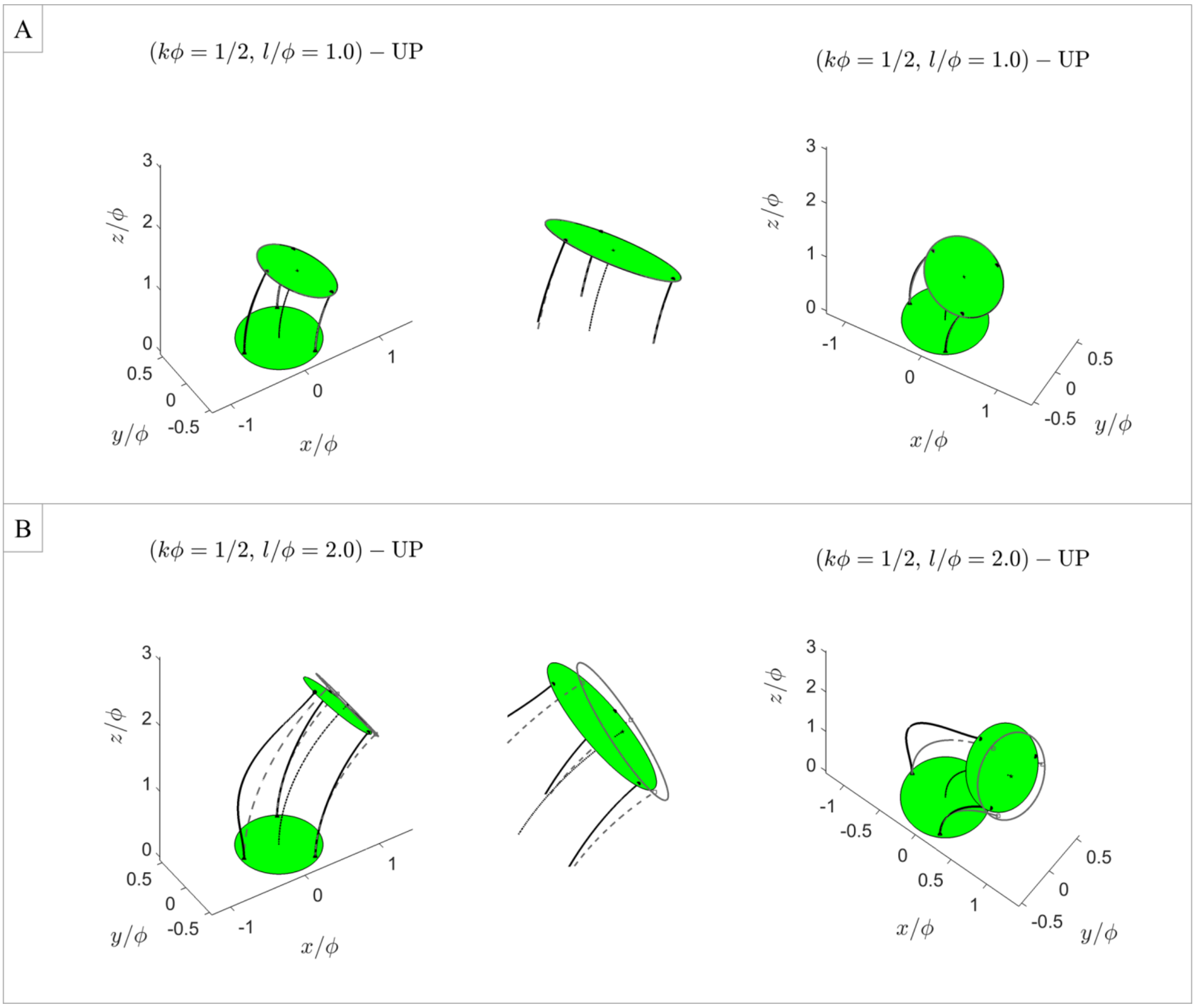

Figure 4. Equilibrium configuration of the idealized probe section, for $\kappa\phi = 1/2$. The UP rod configuration is considered for the LCR, with (A) $\ell = \phi$ and (B) $\ell = 2\phi$. Each subfigure features two views (left and right) of the equilibrium pose, and a detail (middle) of the distal disk. The pursued circular track is also sketched (dashed curves denoting the arcs, the centerline and the distal disk); the FCR rods are not shown for ease of representation. Mechanical instability prevents the probe section in (B) from accurately building the pursued track.

Moreover, Figure 5 shows the trend of the computed deviations, namely δ/ϕ and $\delta\theta$, versus ℓ/ϕ, for selected values of the curvature. In particular, we considered $\kappa\phi = 1/5$, in light of the deployment capabilities stated in [5], as well as the target value $\kappa\phi = 1/2$, to outperform the state of the art. For completeness, we report the trends for both the UP and the FP configuration, the former systematically being the limiting one, as expected. Moreover, we cut the y-axis in the figure in correspondence of the chosen tolerance, namely $\epsilon = 5$ %, to enhance readability. Based on the results in Figure 5, by choosing a probe span $\ell/\phi \cong 1.5$ we should be able to achieve the sought performance, i.e. building a track with a radius of curvature as low as twice the probe diameter. This is the main result of the proposed model: we then addressed the detailed design of the probe by adopting $\ell/\ \phi = 1.5$.

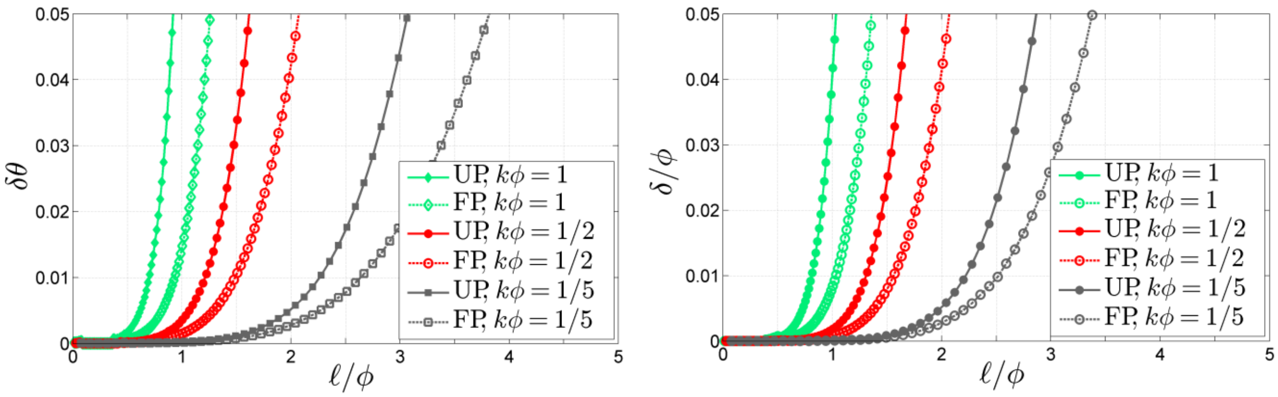

Figure 5. Deviation of the probe section pose (from the pursued circular track) versus ℓ, for selected values of κ. (A) Angular deviation $\delta\theta$. (B) Non-dimensional linear deviation δ/ϕ. Both the FP and the UP rod configurations are considered, for completeness. The $\kappa\phi = 1/2$ curve is the one we targeted for the determination of the probe span. Based on these results, $\ell = 1.5\ \phi$ should permit to achieve the pursued track-building performance, within the chosen tolerance $\epsilon = 5$ %. (The trends associated with the UP configuration are reported in Figure 1D of the main text, for ease of presentation.)

Let us observe that in Figure 5 we also show the trends associated with $\kappa\phi = 1$, just for reference. Indeed, this very high curvature might be achieved e.g. by miniaturized flexible tool tips [13], whereas it is extremely challenging for deployable probes. We did not address this very high curvature at the early developmental stage, because practical implementation constraints (primarily those associated with the non-negligible thickness of the shape-lockers) were likely to hamper the effective achievement of the sought performance. Let us also mention that the computational cost of the curves in Figure 5 was very contained: for any fixed κ and ℓ, it took a few seconds to order of a minute to compute the deviations (elapsed time, on a common desktop PC). Furthermore, those trends in Figure 5 that are associated with the UP configuration are also reported in the main text (Figure 1D), for ease of presentation.

Let us finally remark that, as a byproduct of the developed model, we also obtained a reference value for the rod-clamping force to be achieved through the shape-lockers. In more detail, we firstly computed the maximum tangential force f_{tg} (absolute value) occurring at the endpoints of the LCR rods. For the chosen conditions ($\kappa\phi = 1/2$, $\ell/\phi = 1.5$) we obtained $f_{tg} \cong 5$ N. We then introduced the following inequalities (the approximation symbol reminds that we are dealing with simple estimates):

$$\mu_s\, f_c \gtrsim f_{tg}, \tag{24}$$

$$3\,\mu_s\, f_c \gtrsim 2\, f_{tg}, \tag{25}$$

where f_c denotes the clamping force (absolute value) on a deployment rod, and μ_s indicates the static friction coefficient between the rod and the clamping interface on the shape-locker. In more detail, the condition in Eq. 24 applies to the rod specifically subjected to the extremal value f_{tg}, and it is clearly more stringent than the condition provided by Eq. 25. Differently, the latter condition can be introduced by considering the three rods at once, namely by adding their static friction inequalities (and by assuming that the tangential forces on the two rods that are less loaded sum to f_{tg}, consistently with the equilibrium conditions for the distal disk introduced above). By considering, for instance, a clamping interface made of aluminum, so that $\mu_s \approx 0.45$ [14-16], one obtains $f_c \gtrsim 11.1$ N from Eq. 24 and $f_c \gtrsim 7.4$ N from Eq. 25. Consequently, one should address the detailed design of the shape-lockers by targeting a clamping force around 10 N. We actually considered this reference value since we chose aluminum for the clamping interface, as detailed in the main text.

References

1. Webster RJ, Romano JM, Cowan NJ (2009) Mechanics of Precurved-Tube Continuum Robots. IEEE Transactions on Robotics 25: 67-78.
2. Dupont PE, Lock J, Itkowitz B, Butler E (2010) Design and Control of Concentric-Tube Robots. IEEE Transactions on Robotics 26: 209-225.
3. Xu K, Simaan N. (2006) Actuation compensation for flexible surgical snake-like robots with redundant remote actuation, Proceedings of the IEEE International Conference on Robotics and Automation, Orlando, USA, 4148-4154.
4. Timoshenko S (2001) Theory of elasticity 3rd edition, New York, USA: Ed. McGraw-Hill.
5. Ota T, Degani A, Schwartzman D, Zubiate B, McGarvey J et al. (2009) A Highly Articulated Robotic Surgical System for Minimally Invasive Surgery. Annals of Thoracic Surgery 87: 1253-1256.
6. Gilbert HB, Neimat J, Webster RJ (2015) Concentric Tube Robots as Steerable Needles: Achieving Follow-the-Leader Deployment. IEEE Transactions on Robotics 31: 246-258.
7. Ko SY, Frasson L, Rodriguez y Baena F (2013) Closed-Loop Planar Motion Control of a Steerable Probe With a "Programmable Bevel" Inspired by Nature. IEEE Transactions on Robotics 27: 970-983.
8. Antman SS (2005) Nonlinear Problems of Elasticity 2nd edition, Berlin, Germany: Ed. Springer Science & Business Media.
9. Loeve A, Breedveld P, Dankelman J (2010) Scopes Too Flexible...and Too Stiff. IEEE Pulse 1: 26-41.
10. Bryson C, Rucker C (2014) Toward Parallel Continuum Manipulators. Proceedings of the IEEE International Conference on Robotics and Automation, Hong Kong, China, 778-785.
11. Xu K, Simaan N (2010) Analytic formulation for kinematics, statics, and shape restoration of multibackbone continuum robots via elliptic integrals. Journal of Mechanisms 2: 01100601- 01100613.
12. Webster RJ, Jones BA (2010) Design and Kinematic Modeling of Constant Curvature Continuum Robots: A Review. The International Journal of Robotics Research 29: 1661-1683.
13. Breedveld P, Sheltes JS, Blom EM, Verheij JEI (2005) A new, easily miniaturized steerable endoscope. IEEE Engineering in Medicine and Biology Magazine, IEEE 24: 40-47.
14. Tracton AA (2006) Coatings technology: fundamentals, testing, and processing techniques. Boca Raton, USA: Ed. CRC Press.
15. Davim JP (2012) Tribology in Manufacturing Technology. Berlin, Germany: Ed. Springer Science & Business Media.
16. Davim JP (2012) Tribology in Manufacturing Technology. Berlin, Germany: Ed. Springer Science & Business Media.

[End of Document]
